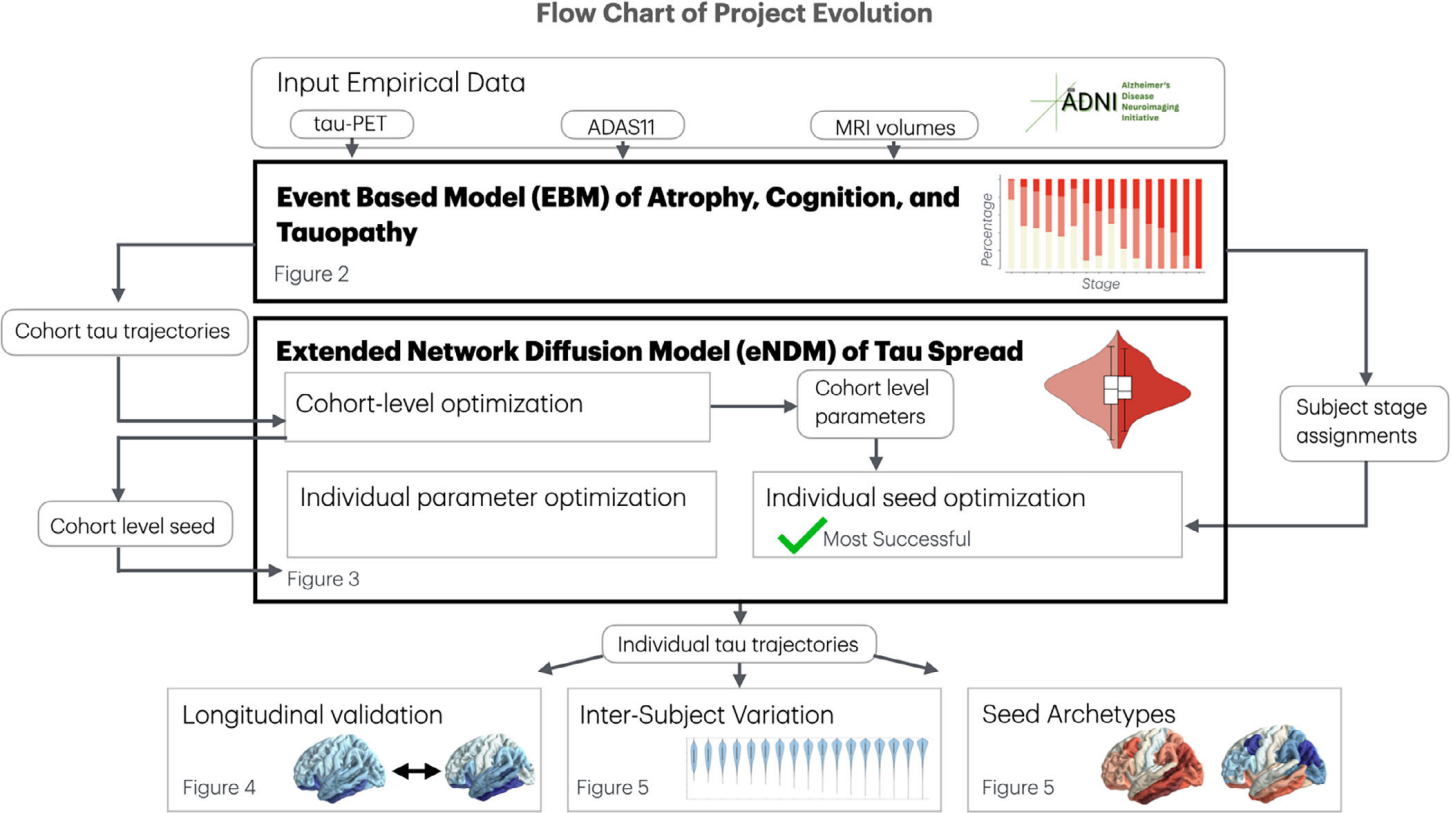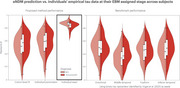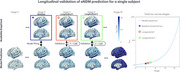# Integrating Event‐Based and Network Diffusion Models to Predict Individual Tau Progression in Alzheimer's Disease

**DOI:** 10.1002/alz70855_107645

**Published:** 2025-12-28

**Authors:** Robin Sandell, Justin Torok, Srikantan Nagaragan, Kamalini G Ranasinghe, Daren Ma, Ashish Raj

**Affiliations:** ^1^ University of California San Francisco, San Francisco, CA, USA; ^2^ UCSF, San Francisco, CA, USA; ^3^ University of California, San Francisco, CA, USA

## Abstract

**Background:**

Alzheimer's disease (AD) affects 55 million people worldwide, with tau protein accumulation following distinct progression patterns. While tau traditionally follows Braak staging, in vivo tau‐PET imaging reveals significant individual variability. Current modeling approaches either lack biophysical basis (statistical Event Based Models ‐ EBMs) or require unavailable longitudinal data (biophysical Network Diffusion Models ‐ NDMs).

**Method:**

We developed a hybrid approach integrating EBMs and NDMs to create predictive individualized models of tau spread. We first applied an EBM to assign disease severity stages to 650 ADNI subjects (64 AD, 196 MCI, 390 controls) based on cross‐sectional biomarker data, producing longitudinal biomarker trajectories from the diverse cohort. We then deployed an extended NDM (eNDM), which models pathological protein spread as a diffusive process on the brain's anatomical network. We fitted the model to the EBM‐derived population‐level trajectories and then to individuals' tau patterns by optimizing both tau's origin, or seed, and kinetic rate parameters controlling network‐based spread and regional accumulation.

**Results:**

Individual seed optimization provided a better fit to subjects' empirical data (mean R=0.85, AIC=9,032) than individual parameter optimization (mean R=0.57, AIC=33,166) and benchmarks from prior studies (mean R=0.36‐0.50). Model predictions were validated by a strong correlation to longitudinal data from 297 subjects with follow‐up tau‐PET scans (mean R=0.81). Analysis revealed that tau patterns show highest heterogeneity at disease onset and converge over time across subjects, challenging conventional assumptions of progressive divergence. Through covariance analysis and clustering, two distinct seeding archetypes were identified: entorhinal‐dominant (typical AD) and diffuse temporal lobe patterns, suggesting multiple pathways of disease initiation.

**Conclusion:**

Our hybrid modeling approach enables individual‐level prediction of tau spread patterns from cross‐sectional data, outperforming previous attempts. Integrating EBMs and NDMs leverages the strengths and tackles the limitations of each technique: EBMs provide longitudinal information, while NDMs generate a biophysical basis for pathology progression. Our findings suggest AD encompasses more diverse tau spread patterns than classical Braak staging, with heterogeneity stemming from distinct seeding sites that converge over time. This framework could guide personalized therapeutic strategies and be applied to other neurodegenerative diseases, potentially improving early diagnosis and treatment selection.